# Socialisation Influences on Gender Ideologies of Immigrant and Native Youth in Germany, England, Sweden and the Netherlands

**DOI:** 10.1007/s11199-020-01208-z

**Published:** 2020-12-06

**Authors:** Laia Sánchez Guerrero, Pia S. Schober

**Affiliations:** 1 Department of Sociology, University of Tübingen, Tübingen, Germany; 2 German Institute for Economic Research (DIW Berlin), Berlin, Germany

**Keywords:** Acculturation, Adolescence, Sex role attitudes, Transgenerational patterns, Migration background, European cultural groups

## Abstract

Adolescence is a crucial life course phase for identity formation, and youths’ gender ideologies significantly predict gendered behaviours and longer-term transitions. With Western post-industrial societies becoming more culturally diverse, the present study provides novel cross-nationally comparative evidence on gender socialisation processes among native and immigrant youth in Sweden, Germany, England, and the Netherlands, which vary in gender and migration policies and cultures. In addition to parents’ gender ideologies, the study also considers classmates’ gender ideologies as factors shaping 14-year-old adolescents’ gender ideologies. The analysis draws on 5917 adolescent-parent dyads from the Children of Immigrants Longitudinal Study which we link with country-level gender empowerment measures from the United Nations. Remarkably, with the exception of native female adolescents in England and Germany, parents tend to report slightly more egalitarian beliefs than adolescents across the four countries. OLS regressions show that parents’ and classmates’ gender ideologies correlate significantly with adolescents’ ideologies, with little variation across gender and immigrant groups in all four countries. From a policy and practical point of view, the great similarity in the intergenerational transmission of gender beliefs across diverse family backgrounds as well as cultural and policy contexts seem remarkable.

**Electronic supplementary material:**

The online version of this article (10.1007/s11199-020-01208-z) contains supplementary material, which is available to authorized users.

Gender egalitarian beliefs and practises have been on the rise in modern post-industrial societies (Bolzendahl and Myers 2004). Yet, some immigrant groups hold more traditional gender beliefs and exhibit larger gender differences in labour market participation and care work than the majority population, with important economic implications for women’s earnings and family income (Arends-Tóth and Van de Vijver 2009; Diehl et al. 2009; Röder 2014; Wang 2019). To elucidate why the gender revolution has unfolded unequally across social groups, we need a more nuanced understanding of how children form their gender beliefs. Youths’ gender ideologies have also been found to significantly predict subsequent family, education, and labour market transitions in adulthood. More egalitarian beliefs among young women have been found to correlate with greater competence beliefs and a higher likelihood of choosing a STEM field of study at university (Van der Vleuten et al. 2016), a greater likelihood of full-time employment and higher hourly wages, less time spent on housework, and later entry into marriage and marital parenthood during adulthood (Corrigall and Konrad 2007; Cunningham et al. 2005; Platt and Polavieja 2016). For young men, egalitarian beliefs during adolescence have been found to predict subsequent preferences and the selection of more stereotypically feminine fields of study at university and a stronger emphasis on social rather than material occupational values (Van der Vleuten et al. 2016) as well as spending more time on housework in adulthood (Platt and Polavieja 2016).

With the populations of European countries becoming culturally more diverse, the present study provides novel cross-nationally comparative evidence on gender socialisation processes during adolescence among native and immigrant parent-child dyads. A number of studies have explored variations in gender beliefs and mechanisms of value transmission from parents to (adult) children in specific immigrant groups within the same country (e.g., De Valk 2008; De Valk and Liefbroer 2007; Goldscheider et al. 2011; Idema and Phalet 2007; Kretschmer 2018). Existing cross-national research on gender beliefs among immigrants has mostly focussed on first- and second-generation adults, without being able to discern intergenerational transmission processes within families (Norris and Inglehart 2012; Röder 2014; Röder and Mühlau 2014). Adolescence is an important life course phase for identity formation (Brechwald and Prinstein 2011) including gender beliefs; however, cross-cultural and institutional influences on gender beliefs among immigrant youth have rarely been investigated. Moreover, even though peer influences on various behaviours have been shown to increase during adolescence (Brechwald and Prinstein 2011), peers’ gender beliefs have not been considered alongside parents’ transmission of values. We thus contribute to the literature by comparing the gender beliefs of immigrant and native adolescents and their parents in four European countries with different gender and migration policies. In addition to parents’ gender ideologies, we also consider classmates’ gender ideologies as a factor shaping the gender beliefs of immigrant as well as native adolescents.

## Gender Socialisation in Immigrant Families

A large number of psychological and sociological studies based on relatively homogeneous samples have generally found moderate associations between parents’ and children’s gender beliefs, with the relations somewhat stronger for mothers than for fathers (for a meta-analysis, see Tenenbaum and Leaper 2002). Recent studies, examining the transmission of gender beliefs by considering both parents’ gender division of labour and gender ideologies, find significant associations between parents’ beliefs and role modelling and children’s later gender beliefs and gender-related work and family aspirations (Crouter et al. 2007; Cunningham 2001; Dawson et al. 2016; Farré and Vella 2013; Platt and Polavieja 2016).

In recent years, acculturation processes concerning gender beliefs among immigrants have received increasing attention. They have been found to vary by immigrant generation, length of residence in the destination country, gender, religious affiliation, social and economic integration as well as the extent to which the gender culture differs between origin and destination country (De Valk 2008; Diehl et al. 2009; Kretschmer 2018; Röder 2014; Röder and Mühlau 2014). Although female immigrants may be more strictly monitored than male immigrants (Idema and Phalet 2007), women are more likely to adopt the values of the receiving country (Idema and Phalet 2007; Röder and Mühlau 2014), possibly due to their stronger interest in and potential gains from gender egalitarianism. Intergenerational stability in gender beliefs is stronger among specific groups of immigrants. For instance, Turkish and Moroccan male immigrants in the Netherlands and Turkish immigrants in Germany have been found to report more traditional work-family plans and gender beliefs than other groups of immigrants. Part of the reason seems to be religious commitments, especially affiliation with Islam, which has been found to correlate with greater preservation of traditional gender beliefs and practises in terms of the division of household tasks (De Valk 2008; De Valk and Liefbroer 2007; Diehl et al. 2009; Kretschmer 2018; Röder 2014). Recent studies also point to discrimination experiences as influential environmental factors which may prompt adolescents to explore their ethnic identity and impact their feelings about their ethnic group membership (Cross 1995; Supple et al. 2006; Umana-Taylor and Guimond 2010).

Adolescence is a critical phase of identity formation during which young people acquire more sophisticated reasoning abilities and are exposed to more diverse ways of doing gender and conceptualising their ethnic group membership in society. A small number of psychological studies has suggested that gender beliefs become more flexible from primary school to early adolescence (Crouter et al. 2007; Dawson et al. 2016; Katz and Ksansnak 1994) but tend to become more traditional again during middle adolescence, which will be the focus of our study. The latter trends may be associated with courtship processes and upcoming occupational choices. For many immigrant adolescents, the process of identity formation involves exploration of their ethnic background, resolution of the personal meaning of their ethnicity, and the affective component of ethnic identity (Phinney and Ong 2007; Umaña-Taylor et al. 2004). Identity formation processes concerning gender and ethnicity are likely to interact in shaping young people’s personal identities and psychosocial development.

In the present study, we examine how variations in parents’ and classmates’ gender ideologies are associated with adolescents’ gender ideologies across immigrant groups in Sweden, Germany, England, and the Netherlands. The four countries show interesting historical variations in their gender equality, migration policies, and the prevalence of choice norms that may shape the intergenerational transmission of gender beliefs among families. It is beyond the scope of our paper to capture the myriad influences of social and economic integration on immigrants’ gender ideologies examined in previous studies. Rather, we focus on parents’ and classmates’ gender ideologies and variations across the gender cultures of origin and receiving countries. We restrict ourselves to exploring gender ideology transmission among classmates rather than close friends or social networks because allocation to classes is usually less selective than children’s choices of close friends.

## Conceptualisation of Gender Socialisation

In contemporary sociology, gender is widely understood as a social structure embedded in the individual, interactional, and institutional dimensions of societies (Risman 2004). Gender identities are reproduced and renegotiated in everyday interactions that are contextually dependent (West and Zimmerman 1987). We define gender ideologies as individuals’ beliefs regarding the appropriate division of paid and unpaid work between men and women (Davis and Greenstein 2009). At the macro level, we refer to gender culture as widespread social beliefs that legitimise or counteract gender inequality (Grunow and Veltkamp 2016).

Socialisation may be understood as the dynamic individual development of a person’s self and personality in constant interaction with the surrounding social structures (Hurrelmann and Bauer 2015). In their social-cognitive theory of gender development, Bussey and Bandura (1999) defined learning processes in gendered social interactions as including direct tutoring, the experience of social sanctions (e.g., praise or disapproval for specific gender-linked behaviours), and role modelling. By observing and interacting with important socialising agents such as parents, whose behaviours provide information on rules and structures of gender-typed activities, children learn which types of conduct are considered gender-appropriate. We assume that children are more likely to form egalitarian gender ideologies when their parents hold more gender-egalitarian ideologies (Hypothesis 1a) and when they practice a more gender-equal division of labour (e.g., with mothers being employed [Hypothesis 1b]).

Concurrently, during adolescence, peers are assumed to be increasingly important in shaping young people’s beliefs and choices. Adolescents invest in peers as sources of social and emotional support and use feedback and acceptance to build a sense of self that gradually becomes more independent of their parents (Prinstein and Dodge 2008). Classmates may shape adolescents’ gender ideologies through value transmission (e.g., by discussing their parents’ division of labour or their own work-family aspirations) and by offering new information and contact to groups that may show more or less gendered behaviours. Classmates are likely to penalise nonconformity and reward conformity regarding gender beliefs, thereby contributing to adolescents’ self-valuation (Brechwald and Prinstein 2011; Lee and Troop-Gordon 2011). We therefore expect that adolescents will hold more gender egalitarian beliefs the more egalitarian their classmates’ ideologies are (Hypothesis 2).

Migration to the four Western post-industrial societies examined in our study—Sweden, England, Germany and the Netherlands—frequently exposes immigrants and their children to a less masculine and hierarchical gender culture within mainstream society compared to their countries of origin. Acculturation refers to “changes in an individual’s cultural patterns (i.e. practices, values, identities) resulting from sustained first hand intercultural contact” (Ward and Geeraert 2016, p. 98). This entails a two-way process of change among minority and majority groups in various values and practice domains, even though most research has focused on adaptation processes for members of minority groups. For the latter, exposure to the majority culture is often assumed to occur via peer socialisation, education, and media consumption. How adaptation unfolds depends inter alia on the importance of specific values within the majority population and how strongly immigrants are expected to integrate in this domain, as well as the extent to which maintaining specific values is perceived as central to the immigrant population’s cultural identity (Drouhot and Nee 2019).

Previous studies have found acculturation among immigrant adults to be more likely or to take place at a faster rate when the gap in gender cultures between the country of origin and receiving country is smaller (Röder 2014; Röder and Mühlau 2014). However, among adolescents, parents are likely to be the main transmitters of the country of origin’s gender culture (Kretschmer 2018). Therefore, the gap in gender cultures between the country of origin and receiving country may not directly influence adolescents’ gender beliefs when their parents’ gender ideologies are taken into account. However, this gap may well act as a moderating factor, because immigration, particularly from countries with more traditional gender and family cultures, might strengthen family ties and the importance of family values in the receiving country (Pels and Nijsten 2003). Simultaneously, exposure to a very different gender culture in school and the media might challenge existing family relations and weaken intergenerational transmission (Phalet and Schönpflug 2001). To date, a few studies that were able to draw on parent-child dyads (De Valk 2008; De Valk and Liefbroer 2007) found the intergenerational transmission of work-family preferences to be similar among four immigrant groups in the Netherlands compared to the Dutch population, possibly due to offsetting effects of these opposing mechanisms. Therefore, we formulate two competing hypotheses: (a) Immigrant parents’ gender ideologies will be more strongly associated with adolescents’ when the gender culture of the country of origin is more dissimilar to that of the receiving country (i.e., cultural difference strengthens intergenerational transmission; Hypothesis 3a) and (b) Immigrant parents’ gender ideologies will be more strongly associated with adolescents’ when the gender culture of the country of origin is more similar to that of the receiving country (i.e., cultural difference weakens intergenerational transmission; Hypothesis 3b).

## Gender Culture of the Receiving Countries

In the present study we conceptualise state policies as actively creating and reinforcing gender and immigration cultures (Chang 2000; Grunow and Veltkamp 2016; Kremer 2007; Wimmer and Soehl 2014). Because education programmes aiming to reduce gender gaps (OECD 2015) have been implemented only relatively recently and have not been systematically compared across countries, we draw on long-standing variations in family and labour market policies which shape gender cultures. In addition, antidiscrimination, multicultural, and citizenship policies are assumed to influence adolescents’ gender ideologies and the extent to which immigrant parents transmit their own beliefs to their children. The four countries in our study—England, the Netherlands, Sweden, and Germany—show interesting variations in their supports for working mothers and for involved fathers and in their antidiscrimination, multicultural, and citizenship policies.

Whereas Sweden has long combined a formal commitment to gender equality with strong provision of support services for working mothers and caring fathers (Goldscheider et al. 2011), England and the Netherlands have implemented equal pay and antidiscrimination laws to provide women and men with equal access to the labour market but do not actively support gender equality in the private sphere (Chang 2000; Grunow and Veltkamp 2016). In line with the traditional family-centred regime, public policies in West Germany long encouraged women to structure their employment around family obligations (e.g., by taking long leaves after childbirth) and provided little childcare support. Despite a paradigm shift since the mid-2000s (Stahl and Schober 2018), children born in the 1990s were probably still influenced by Germany’s strong familialist norms at the time. Immigrant parents’ influence on their children’s beliefs and aspirations is likely to be weaker in contexts with a strong normative culture, such as Sweden’s institutionalized gender equality culture or Germany’s familialist system (Holland and de Valk 2017). By contrast, intergenerational transmission is likely to be stronger in England and the Netherlands where policies do not clearly support any single family model and where norms of choice have been on the rise (Knight and Brinton 2017). According to this view, gender-role differences are acceptable as long as they arise from autonomous individual choices (Knight and Brinton 2017).

The four countries also vary in their antidiscrimination, multicultural, and citizenship policies (Drouhot and Nee 2019; Koopmans 2013). Several studies have argued that legal or economic disadvantages experienced by immigrant groups are likely to slow down acculturation, which in such cases offers smaller returns (Wimmer and Soehl 2014). Sweden, England, and the Netherlands developed antidiscrimination and multicultural policies during immigration waves between the 1950s and 1980s. Naturalisation policies varied and were most generous in England and the Netherlands for immigrants from former colonies. Germany framed most of the migrant population as guest workers, pursued very restrictive citizenship and multicultural policies, and introduced antidiscrimination policies only in 2006 (Drouhot and Nee 2019; Joppke 2007; Koopmans 2013). Germany’s restrictive policies (Koopmans 2013) are likely to slow down acculturation among most groups of immigrants more than the multicultural policies in the other countries.

In conclusion, due to Sweden’s strong normative culture of gender equality paired with antidiscrimination policies and fairly liberal citizenship laws, we expect immigrant youth to hold more egalitarian gender ideologies in Sweden than in the other three countries, even after controlling for differences in gender culture between the receiving and origin countries (Hypothesis 4). Given that it is unclear whether the legal disadvantages faced by most immigrant groups in Germany will outweigh the effects of weaker gender norms in England and the Netherlands, we do not predict a ranking of these countries. For the same reasons, we assume that immigrant parents’ gender ideologies will be less strongly associated with adolescents’ ideologies in Sweden than in Germany, England, and the Netherlands (Hypothesis 5).

## Method

### Data

We use the first wave of the Children of Immigrants Longitudinal Survey in Four European Countries (CILS4EU). In 2010, the CILS4EU study (Kalter et al. 2017a) sampled about 500 schools and interviewed more than 18,000 pupils in eighth grade (14-year-olds), as well as their parents and teachers, across England, Germany, the Netherlands, and Sweden. In total, 10,663 parents and 18,702 adolescents answered the questionnaires. Only one parent of each adolescent was interviewed; 79% of those surveyed were mothers. In some countries, tracking systems result in some of the sampled youth changing or leaving schools in the second wave. This tracking effect produced a substantial loss of sample in the Netherlands, with new adolescents from the resulting classes being added in Wave 2. Because this change complicates the estimation of classmate effects in Wave 2, we focus our analysis on Wave 1 for all countries. Due to its nested class and school structure and cross-nationally comparative design, the dataset is well suited to investigate the relationships among adolescents’ gender ideologies and those of their parents and classmates among large samples of native and immigrant youth.

### Sample Selection and Nonresponse

The analytical sample included all adolescents and parents for whom we had information on their immigrant background and gender ideologies. Hence, we have information about 204 immigrants and 622 natives in England, 879 immigrants and 1250 natives in Germany, 268 immigrants and 1375 natives in the Netherlands, and 285 immigrants and 1034 natives in Sweden. In England, a significant share of immigrants in the sample are from former colonies, specifically India (21%), followed by Pakistan (17%), Ireland, and Eastern Africa (each around 7%). In Germany, immigrants from Turkey (38%), Eastern Europe (35%), and Southern Europe (10%) make up the largest groups. In the Netherlands, most immigrant youth have a Turkish (15%), Moroccan (7%) Surinamese (10%), and European background (27%). In Sweden, the largest ethnic minority groups are from other Nordic countries (21%), Eastern Europe (21%) and the Middle East (17%). The immigrant populations in the four countries are not only diverse in terms of origin but also regarding various aspects of economic and social integration and religion (for details, please see De Valk 2008; De Valk and Liefbroer 2007; Dustmann and Frattini 2011; Filipp et al. 2016; OECD 2014).

In sum, we have information for 1636 first- or second-generation immigrant adolescents and their parents and 4281 natives or third-generation (or above) immigrant adolescents and their parents. In total, these adolescents are nested in 884 classes, 464 schools, and four countries. We applied design and wave nonresponse weights to account for differential nonresponse and the overrepresentation of immigrants in the sample. Because schools with a large share of immigrant adolescents were oversampled by design, CILS4EU provides design weights to correct for the different selection probabilities of schools and classes due to unequal ethnic density among schools. In addition, these design weights are combined with non-response adjustment weights to account for various characteristics of non-responding schools, classes, and students in the sample. (For more detailed information about how these weights were calculated, please see CILS4EU 2016). In the end, the weighted data should be roughly representative of the overall student population within each of the four countries.

Item non-response for the questions measuring the gender ideologies of youth and their parents was modest, at only 3% and 5% respectively. The variables with the largest share of missing responses (about one quarter) related to sibling composition and paternal education. We used multiple imputation by chained equations with five imputation cycles to impute the missing observations for the individual-level independent variables. Because the results with imputed data were very similar to those based on the non-imputed data, the latter are presented in the results section. Slight differences that emerged are discussed in the sensitivity analysis.

### Analytical Strategy

We applied ordinary least squares regression (OLS) models with standard errors clustered at the school level. Despite the nested structure of the dataset, we used OLS models rather than multilevel modelling because the nonsignificant intraclass correlation suggested that OLS was a better fit for the analysis. We ran separate models by gender and immigrant background because previous studies pointed to varying mechanisms across groups and we wanted to be able to consider immigrant-specific variables. The immigrant subsamples were too small to allow for cross-national comparisons of immigrant youth from specific countries of origin across two or more receiving countries. Therefore, we followed the approach by Röder and Mühlau (2014) and matched individual-level information on origin and destination country with the country-level gender empowerment measure (GEM) developed by the United Nations Development Program*.* This procedure allows us to consider variations in the gender culture of immigrants’ countries of origin as well as across the four receiving countries.

### Dependent Variable: Gender Ideologies

To capture youths’ gender ideologies concerning the appropriate gender division of paid and unpaid labour, we drew on four items. CILS4EU asked adolescents who should perform the following four tasks in a family: (a) take care of children, (b) cook, (c) clean the house, and (d) earn money. Respondents were given three possible answer categories: (1) mostly the man, (2) mostly the woman, and (3) both about the same. Because very few adolescents chose the non-traditional categories (e.g., mostly the man should clean the house), we recoded the original variables into four dummy variables distinguishing a more egalitarian gender ideology from beliefs favouring a traditional gender division of labour. (Descriptive statistics for individual items are shown in Table 1s in the online supplement.) We then applied a polychoric factor analysis (α = .75) to construct a combined factor, with higher values indicating more egalitarian gender ideologies (actual values ranged from 0 to 1.07). For the regressions, we *z*-standardised this variable.

As shown in Table 1, female adolescents held more egalitarian beliefs on average than male adolescents across countries and among both natives and immigrants. Native adolescents held more egalitarian beliefs than their immigrant counterparts. Finally, both immigrants and natives report the most egalitarian beliefs in Sweden, followed by England. The difference between Germany and the Netherlands was small, with German natives reporting more egalitarian ideologies than Dutch natives, whereas for immigrants the relationship was reversed.

**Table 1:** Descriptive statistics for adolescents’ gender ideologies by immigrant status and adolescents’ gender (weighted)

	Immigrant		Natives	
	Female	Male	Female	Male
Destination country	*M* (*SD*)	*M* (*SD*)	*M* (*SD*)	*M* (*SD*)
England	.661 (.389)	.594 (.415)	.815 (.343)	.636 (.397)
Germany	.572 (.369)	.455 (.362)	.710 (.348)	.556 (.394)
Netherlands	.664 (.317)	.523 (.390)	.680 (.367)	.531 (.373)
Sweden	.897 (.285)	.788 (.368)	.985 (.206)	.845 (.334)

Higher values represent more egalitarian ideologies. All gender differences within immigrant background are significant (*p*s < .01). All immigrant/native background differences within each gender are significant (*p*s < .01). All country differences are statistically significant at the *p*s < .01, with the exception of those between the Netherlands and Germany, which are statistically significant at the *p*s < .05 level. Source: CILS4EU – Full version (ZA5353 data file version 3.3.0)

### Explanatory Variables

#### Parental Gender Ideologies

Using the same items as for adolescents, CILS4EU asked parents who should (a) take care of children, (b) cook, (c) clean the house, and (d) earn money. Following the same steps as for youth gender ideologies, we constructed a parental gender ideology factor by conducting a polychoric factor analysis (α = .77). To facilitate comparability between parents’ and adolescents’ ideologies, we held the scoring coefficients constant across both variables by using those for the adolescents’ gender ideology indicator. Remarkably, parents tend to report slightly more egalitarian beliefs than their children across all countries (see items in Table 1s in the online supplement).

#### Class Gender Ideologies

We consider the mean gender ideology among classmates by summing up the gender ideology scores of the respondent’s classmates who participated in the CILS4EU and dividing them by the number of classmates interviewed.

#### Mother’s and father’s Employment Status

We included two dummy variables to account for the employment status of the mother and father, respectively. These variables take the values (1) employed or (0) not employed. Unfortunately, information about working hours, part-time employment or the division of household labour was not available.

#### Immigrant Generation

We categorised adolescents as immigrants if they were first- or second-generation immigrants, with the latter consisting of native-born adolescents with one or both parents born abroad. Previous research has shown that by the third generation, most immigrant groups tend to behave very similarly to natives in many domains (Heinrich-Böll-Stiftung 2010; Logan and Shin 2012). Further tests on our data likewise do not show significant differences in average gender ideologies between third-generation youth compared to natives. In the models for immigrant youth, we included another dummy variable that takes a value of 1 if the child is a second-generation immigrant (80%) and 0 if he or she is a first-generation immigrant (20%).

#### Difference in Gender Empowerment Measure between Origin and Destination Country

As a proxy for cross-national differences in gender culture, we draw on the GEM developed by the United Nations Development Program to capture the diversity of origin countries of immigrants in the Netherlands, England, Sweden, and Germany. This measure is based on estimates of women’s relative income, attainment of high-paying positions, and attainment of professional and parliamentary positions. Compared to alternative measures, such as the UN Gender Inequality Index or the female-male labour force participation ratio, the GEM was more strongly correlated with adolescents’ gender ideologies. Higher GEM values imply greater gender empowerment.

To account for gender culture distance between the destination and origin countries, we subtracted the 2009 GEM value of the origin country from that of the destination country. Because not all countries of origin in the sample had GEM values available, missing values were imputed using their regional averages. Because previous studies provide mixed evidence as to whether gender ideologies are transmitted equally by both parents, more strongly by mothers, or by the same-sex parent (Dawson et al. 2016; Idema and Phalet 2007; Tenenbaum and Leaper 2002), we assign the average gender culture of their parents’ countries of origin for adolescents with parents of mixed origin for simplicity’s sake. The resulting index was recoded into tercile categories corresponding to (1) small, (2) medium, and (3) large differences. For illustration, the modal countries of origin in each category for each destination country are as follows: For England, Poland (small), Pakistan (medium), and Bangladesh (large) are the most common countries of origin in each category. In Germany, these are Poland (small), Russia (medium), and Turkey (large); in the Netherlands, they are Germany (small), Suriname (medium), and Morocco (large). Finally, in Sweden, Germany (small), Poland (medium) and Iraq (large) are the most common countries of origin in each category.

### Control Variables

#### Adolescents’ Contact with Natives

Studies on inter-ethnic friendships point to social integration in the sense of social contacts and affective closeness with members of the mainstream society as an important driver of value adoption (Goldscheider et al. 2011; Kretschmer 2018). We constructed an indicator of contact with natives by implementing a polychoric factor analysis of two items: (a) how much time adolescents spent in the neighbourhood with natives and (b) how many of their friends were natives. Answers for the former item range from 1 (*I do not know people of this background*) to 6 (*every day*). Answers for the latter item range from 1 (*none or very few*) to 5 (*almost all or all*). The resulting factor variable, with higher values indicating more frequent contact with natives, was then standardised for the regression models. Still, despite the high correlation between the two items (*r* = .50), the combined factor had only a moderate alpha value of .65. Thus, we alternatively tested whether the results changed if we used only the second item as an indicator of adolescents’ social integration with natives. To facilitate the interpretation of this individual item, we aggregated the five original categories into the following three categories: (1) none or few, (2) half or a lot, and (3) all or almost all. (Descriptive statistics for both the factor variable and the second item can be found in Table 2s in the online supplement.)

#### Parents’ Contact with Natives

Because cohesive social networks among immigrant families are likely to slow down the acculturation process, we also account for the extent to which parents are socially integrated with natives by using parents’ responses to the same questions as for adolescents. We again applied polychoric factor analysis, and the variable was standardised for the analysis. Once again, despite the two items’ relatively high correlation (*r* = .53), the alpha was moderate (.56). For this reason, we also tested whether our results changed if we used just the second of these items capturing parents’ friendships with natives. This variable was recoded into the same three answer categories as for adolescents and included as an alternative control variable.

#### Religiosity and Religious Denomination

Because some previous studies point to religious beliefs as important factors in gender ideology formation (Diehl et al. 2009; Kretschmer 2018), two measures were included to account for the effects of religion. First, we applied polychoric factor analysis (α = .83) to capture adolescents’ religious commitment based on three questions: (a) how often the adolescent visits a religious meeting place (answers range from 1 “never” to 5 “every day”), (b) how often s/he prays (answers ranging from 1 “never” to 6 “five times a day or more”), and (c) how important religion is to her/him (answers range from 1 “not at all important” to 4 “very important”). Second, we accounted for the respondents’ religious denomination. For immigrants, we distinguished between (1) no religion, (2) Christianity, (3) Islam, and (4) other*,* which includes Judaism, Buddhism, Hinduism, Sikhism, and other religions. For natives, Islam is included in the category “other” due to very few cases of Muslim natives.

#### Mother’s and Father’s Education

Because economic integration has been shown to impact intergenerational value transmission (Wimmer and Soehl 2014), we considered two dummies accounting for whether each parent holds a tertiary degree (0 = no; 1 = yes). The way the information was collected in the Netherlands made a more detailed harmonisation with the other three countries very difficult.

#### Family Structure

Because some studies have found that children living with a single mother hold less traditional identities (Stevenson and Black 1988) and the influence of step-parents on children’s gender ideologies and choices tends to be weaker (Carlson and Knoester 2011; Laftman 2008), we constructed a categorical variable indicating whether the adolescent lived (1) with both their biological or adoptive parents, (2) in a stepfamily, or (3) with a single parent. Having a sibling of the other gender may affect adolescents’ gender ideology formation. Unfortunately, we were not able to account for the number of sisters and brothers, but we included two dummy variables accounting for the absence (0) or presence (1) of at least one brother and at least one sister in the household, respectively. We controlled for the number of people living in the primary household reported by the adolescent.

#### Class and School Composition

Because 14-year-old adolescents have already been sorted into different educational tracks in Germany and the Netherlands but not in England and Sweden, and these tracks tend to be segregated by gender and migration status, we controlled for the gender composition of the class and the share of immigrants in the school’s student body. The latter variable, provided by CILS4EU, consists of five categories (1) 0–10%; (2) 10–30%, (3) 30–60%, (4) 60–100%, and (5) independent schools, which did not provide this information.

#### Gender of Respondent’s Parent

We included a dummy variable to account for any differential effects of the gender of the responding parent (0 = father; 1 = mother). Because some previous studies point to stronger belief transmission by mothers than by fathers (Idema and Phalet 2007; Tenenbaum and Leaper 2002), we also tested for interactions between parents’ gender ideologies and the gender of the respondent parent; however, these proved to be statistically nonsignificant.

## Results

Table 2 shows the descriptive statistics of our key explanatory variables. On average, both native parents and adolescents report more egalitarian ideologies than their immigrant counterparts. Perhaps due to segregation in the school system, natives attend school classes where students report on average slightly more egalitarian ideologies than immigrants. Whereas the vast majority of mothers in our sample are employed, there are substantial differences between native and immigrant mothers: 88% of native mothers are in paid employment whereas this applies to only 68% of immigrant mothers.

**Table 2:** Descriptive statistics (weighted)

	Immigrants	Natives
Variables	*M* (*SD*) or *n* (%)	*M* (*SD*) or *n* (%)
Adolescent’s gender ideology^a^	.60 (.39)	.70 (.37)
Parental gender ideology^a^	.69 (.37)	.83 (.359)
Mean class gender ideology^a^	.63 (.15)	.69 (.15)
Mother employed		
No	524 (32)	514 (22)
Yes	1112 (68)	3767 (88)
Distance in gender empowerment between origin and destination country		
Small	539 (33)	-
Medium	556 (34)	-
Large	540 (33)	-

Source: CILS4EU – Full version (ZA5353 data file version 3.3.0)

^a^Higher values indicate more egalitarian gender ideology

Table 3 shows the results of OLS regressions with adolescents’ gender ideologies as the dependent variable separated by gender and immigrant background. We observe significant associations with the gender ideology of the respondent parent for all subgroups. An increase in parents’ egalitarianism of one standard deviation is associated with a change in adolescents’ egalitarianism of about a quarter of a standard deviation. The strength of the relationship does not differ substantially by gender or by immigrant background, as shown by additional tests with gender interaction terms in the two models for immigrant and native youth, respectively (see Table 5s in the online supplement). These results support Hypothesis 1a, which stated that adolescents hold more egalitarian beliefs about the gender division of labour when their parents also hold more egalitarian gender ideologies.

**Table 3:** OLS regression for adolescents’ gender ideologies by gender and immigrant background

		Immigrants				Natives			
		Female (*n* = 842)		Male (*n* = 794)		Female (*n* = 2,169)		Male (*n* = 2,112)	
Variables		*b*	95% CI	*b*	95% CI	*b*	95% CI	*b*	95% CI
Parental gender ideology^a^		.27***	[.20, .34]	.24***	[.16, .33]	.26***	[.22, .31]	.23***	[.19, .28]
Class gender ideology^a^		.12**	[.04, .20]	.05	[−.04, .13]	.08***	[.04, .13]	.09***	[.04, .14]
Mother employment^a^		.18*	[.02, .34]	.28***	[.14, .45]	.29***	[.17, .40]	.37***	[.21, .50]
Distance in gender empowerment									
(Ref. Small)									
Medium		−.07	[−.23, .09]	−.15	[−.32, .03]	--	--	--	--
Large		−.09	[−.30, −13]	−.07	[−.25, .12]	--	--	--	--
Destination country									
(Ref. Sweden)									
England		−.14	[−.43, .14]	−.14	[−.43, .15]	−.15*	[−.27, −.02]	-.22**	[-.38, -.05]
Germany		−.31**	[−.51, −.12]	−.46***	[−.68, −.24]	−.28***	[−.39, −.17]	-.30***	[-.47, -.14]
Netherlands		−.31**	[−.56, −.06]	−.42***	[−.68, −.17]	−.33***	[−.46, −.20]	-.37***	[-.54, -.22]
Constant		.57*	[.07, 1.08]	.16	[−.36, .67]	.45***	[.16. .75]	.03	[-.36, .43]
*R*^2^		.28		.24		.26		.20	

*z*-transformations of factor variables; Ref.= Reference category. Control variables: Mother's education, father's education, second generation immigrant, youth integration with natives, parent integration with natives, religion, religiosity, family structure, sister dummy, brother dummy, household size, respondent is the mother, share of immigrants in school, share of females in class. The coefficients for the control variables are available in Table 2s in the online supplement. Source: CILS4EU – Full version (ZA5353 data file version 3.3.0)

^a^Higher values indicate more egalitarian gender ideology

*p < .05. ** *p* < .01. *** *p* < .001

Additional models with an interaction term between parents’ gender ideologies and the gender of the respondent parent did not reach statistical significance (see Table 5s in the online supplement). It is also noteworthy that, after considering parents’ gender ideologies, the GEM distance between origin and receiving country is not statistically significant among immigrant adolescents, irrespective of gender. In line with Kretschmer (2018), parents appear to constitute the main transmission channel of origin country’s gender culture. In line with Hypothesis 1b, adolescents whose mothers were employed reported significantly more egalitarian ideologies, with associations varying between 18% and 37% of a standard deviation. Again, additional tests in a joint model with interaction effects revealed nonsignificant differences in the strength of the relationships with maternal employment (see Table 5s). Given that we have no information as to whether the mother worked full-time or part-time, the effects may vary depending on the level of maternal labour force engagement.

The positive relationships with classmates’ gender ideologies turn out to be weaker than those with parents’ gender ideologies, albeit still statistically significant for all groups except immigrant male adolescents. The associations range from 12% of a standard deviation for female immigrants to 5% for male immigrants. However, further tests with interaction effects in joint models show that these small differences across subgroups were not statistically significant (see Table 5s in the online supplement). These results mostly support Hypothesis 2, suggesting that classmates significantly shape the gender ideologies of immigrant as well as native adolescents.

To uncover whether the strength of the association with parental gender ideology differs depending on the gender empowerment cultural distance between the receiving and origin country (Hypotheses 3a and 3b), we interacted parental gender ideology with GEM distance. The interaction is negative but only close to statistically significant for male immigrants (*p* = .06) and not significant for female immigrants (see Table 4a). On the whole, therefore, we reject Hypotheses 3a and 3b because the relationships between parents’ and adolescents’ gender ideologies is relatively similar irrespective of the difference in gender empowerment culture between the receiving and origin country. Additional sensitivity tests with a combined sample of male and female immigrants show no substantial differences in these results (see Table 6s in the online supplement).

**Table 4:** Main effects of parental gender ideology and interactions for immigrant adolescents’ gender ideologies

	Females (*n* = 842)		Males (*n* = 794)	
Variables	*b*	95% CI	*b*	95% CI
(a) Hypotheses 3a and 3b: Interaction between parental gender ideology and similarity of gender culture				
Parental gender ideology^a^	.27***	[.17, .38]	.32***	[.21, .44]
Distance in gender empowerment (Ref. low)				
Medium	−.07	[−.23, .09]	−.16	[−.34, .01]
Large	−.10	[−.34, .13]	−.12	[−.32, .08]
Interaction				
Parental gender ideology^a^ x distance in gender empowerment (Ref. Small)				
PGI x Medium	.00	[−.15, .16]	−.11	[−.27, .03]
PGI x Large	−.04	[−.19, .13]	−.15	[−.31, .00]
Constant	.56**	[−07, 1.07]	.19	[−.33, .72]
*R*^2^	.27		.24	
(b) Hypothesis 5: Among immigrants, parents’ gender ideologies are less strongly associated with children’s ideologies in Sweden than in Germany, England, and the Netherlands				
Parental gender ideology^a^	.15	[−.01, .32]	.19	[−.01, .40]
Destination country (Ref. Sweden)				
England	−.14	[−.45, .17]	−.09	[−.39, .20]
Germany	−.34***	[−.55, −.14]	−.47***	[−.70, −.25]
Netherlands	−.33**	[−.59, −.08]	−.44***	[−.71, −.18]
Interaction				
Parental gender ideology^a^ x destination country (Ref: Sweden				
PGI x England	.16	[−.09, .43]	.20	[−.09, .50]
PGI x Germany	.10	[−.07, .29]	.02	[−.20, .25]
PGI x Netherlands	.14	[−.06, .35]	−.00	[−.27, .24]
Constant	.60**	[.09, 1.11]	.16	[−.35, .68]
*R*^2^	.27		.24	

*z*-transformations of factor variables; Ref.= Reference category. Source: CILS4EU – Full version (ZA5353 data file version 3.3.0)

^a^Higher values indicate more egalitarian gender ideology

*p < .05. ** *p* < .01. *** *p* < .001

Hypothesis 4 assumed that the gender ideologies of immigrant youth would be more egalitarian in Sweden than in the other three countries. The main effects of the country dummies (see Table 3) show that immigrant youth in Sweden held significantly more egalitarian ideologies than those in Germany and the Netherlands. The differences between England and Sweden were not statistically significant for immigrant youth, whereas they were for natives. The differences between Germany and the Netherlands were negligible in size and not statistically significant. These results therefore provide partial support for the influence of the gender culture of the destination country, in line with Hypothesis 4.

Finally, we have to reject Hypothesis 5 because parental gender ideologies were not less strongly associated with immigrant adolescents’ gender ideologies in the strong normative egalitarian context of Sweden compared to the other three countries. The interaction effects between receiving country and parental gender ideologies were not statistically significant (see Table 4b). Separate regression models for the four countries also suggested that the associations with parental gender ideologies were very similar across countries (available from the authors upon request). (The results for the control variables are shown in Table 3s in the online supplement.)

We have conducted a number of sensitivity analyses to test the robustness of our results. Multicollinearity tests using the variance inflation factors did not suggest any issues. In addition, as shown in Table 5, the vast majority of correlations between the variables in the model are moderate. Multiple imputation models using chained equations came to generally very similar results (available from the authors upon request). In a very small number of instances, the associations were slightly more significant in the imputed models, such as the effects of average class gender ideology for male immigrants.

**Table 5:** Pairwise correlation matrices

Variables	Adolescent gender ideology	Parent gender ideology	Class gender ideology	Female	University-educated mother	Religiosity	Religion
Parental gender ideology	.38	--	--	--	--	--	--
Class gender ideology	.33	--	--	--	.32	--	--
Mother employment	--	.31	--	--	--	--	--
University-educated mother	--	--	.32	--	--	--	--
University-educated father	--	--	--	--	.45	--	--
Religion	--	--	--	--	--	.52	--
Religiosity	--	--	--	--	--	--	.52
Adolescent contact with natives	--	--	--	--	--	--	--
Adolescent proportion of native friends	--	--	--	--	--	--	-.35
Parent contact with natives	--	--	--	--	--	--	-.33
Parent proportion of native friends	--	--	--	--	--	-.31	-.38
Immigration background	--	--	--	--	--	.33	.38
Household size	--	--	--	--	--	--	--
Share of females in school	--	--	--	.38	--	--	--
Share of immigrants in school	--	--	--	--	--	--	.30
Germany	--	--	-.41	--	--	--	.31
Sweden	.31	--	.52	--	.37	--	--
	Adolescent contact with natives	Parent contact with natives	Gender empowerment distance	Adolescent proportion of native friends	Parent proportion of native friends	Sister dummy	Brother dummy
Parental gender ideology	--	--	--	--	--	--	--
Class gender ideology	--	--	--	--	--	--	--
Mother employment	--	--	--	--	--	--	--
University-educated mother	--	--	--	--	--	--	--
University-educated father	--	--	--	--	--	--	--
Religion	-.35	-.33	.35	-.35	-.38	--	--
Religiosity	--	--	.42	--	-.31	--	--
Adolescent contact with natives	--	.43	-.31	.78	--	--	--
Adolescent proportion of native friends	.78	.36	-.30	--	.41	--	--
Parent contact with natives	.43	--	-.30	.36	--	--	--
Parent proportion of native friends	--	--	-.35	.41	--	--	--
Immigration background	-.33	-.37	--	-.35	-.45	--	--
Household size	--	--	--	--	--	.39	.35
Share of females in school	--	--	--	--	--	--	--
Share of immigrants in school	-.41	--	--	-.41	--	--	--
Germany	--	--	--	--	--	--	--
Sweden	--	--	--	--	--	--	--

Only correlations greater than or equal to |.30| are included. Source: CILS4EU - Reduced version (ZA5656 data file *Version* 3.3.0)

Stepwise models showed that entering the control variables hardly affected the results presented here for the key explanatory variables. In addition to maternal employment status, we also tested associations with the gender typicality of mothers’ and fathers’ occupations, measured as the share of women in each occupation, and we did not find significant associations with adolescents’ gender ideologies.

To reduce the risk of reverse causality, with adolescents also potentially influencing their parents’ ideologies, we reran all models with the dependent variable of adolescents’ gender ideologies measured in Wave 2 instead of Wave 1 (Kalter et al. 2017a). The results of these regressions show slightly weaker but still statistically significant associations between parents’ and adolescents’ gender ideologies (available from the authors upon request).

To test whether our statistically significant associations were the product of error inflation due to splitting the sample into four groups, we ran the OLS regression models with Bonferroni corrections for immigrant and native students, with gender as a control variable (see Table 4s in the online supplement) and additional interaction terms between our key variables and gender (see Table 5s in the online supplement). Following the same approach, we also applied Bonferroni corrections to the models with interaction effects testing Hypotheses 3a and 3b and Hypothesis 5 (see Table 6s in the online supplement). Finally, because the alphas of the two-factor variables capturing adolescents’ and parents’ social integration with natives were moderate, we re-ran the models with two single items measuring parents’ and adolescents’ proportion of native friends, respectively, instead of the aforementioned factor variables. Altogether, the very similar results of all these models show that our findings are robust. (It is important to note that the robustness checks shown in Tables 4s, 5s, and 6s in the online supplement had to be performed using the Scientific Use File (Kalter et al. 2017b) instead of the Restricted Full Version of the CILS4EU (Kalter et al. 2017a) because the latter can only be analysed in a secure data centre, which was under lockdown at the time of writing due to the Covid-19 pandemic.) The only difference relevant for our analysis is that in the Scientific Use File, the country of origin is presented in more aggregated regional categories for countries with only a small number of observations (e.g., Southern Europe instead of Spain). Therefore, the GEM difference between origin and receiving country for the robustness checks was constructed by linking the UN Gender Empowerment Measure to this region of origin information.

## Discussion

The present study advances our understanding of how adolescents of varying cultural origins form their gender ideologies regarding the appropriate gender division of paid and unpaid work. First, the paper elucidates the intergenerational transmission of gender ideologies across immigrant and gender groups while considering differences in gender empowerment cultures among a diverse set of origin countries and the four receiving countries. Second, we provide novel evidence on the role of parents and classmates in shaping youths’ gender ideologies. Overall, we find that parents’ and classmates’ gender ideologies correlate significantly with adolescents’ ideologies, with little variation across gender and immigrant groups, in all four countries.

Across all four countries included in our analysis, native female adolescents report the most egalitarian ideologies, followed by immigrant female adolescents and native male adolescents, whereas immigrant male adolescents generally hold the most traditional gender beliefs. Across all four groups, gender ideologies of parents, mostly mothers, correlate significantly and strongly with adolescents’ ideologies. The similar strength of association between immigrants and natives is in line with findings from two Dutch studies on young adults (De Valk 2008; Huschek et al. 2010). Previous studies (Pels and Nijsten 2003; Phalet and Schönpflug 2001) observed two opposing mechanisms for immigrant adolescents: (a) a stronger gender culture transmission due to closer family bonds or (b) a weaker transmission due to exposure to a different gender culture in the receiving country. Our findings indicate a relatively similar intergenerational transmission among immigrant youth irrespective of gender-cultural background. One possible explanation may be that the two opposing mechanism offset each other, leading to nonsignificant variations. However, it should be noted that our analysis mostly captures the transmission of mothers’ gender ideologies. Hence, we cannot exclude that the transmission of fathers’ beliefs may be stronger, especially for immigrants from countries where the gender culture tends to be less empowering for women. Unfortunately, our data did not allow us to capture both parents’ gender ideologies.

In line with a large branch of the literature on gender socialisation through role modelling (e.g., Cunningham 2001; Dawson et al. 2016; Farré and Vella 2013; Platt and Polavieja 2016), maternal employment proved to be a significant and relatively strong predictor of more egalitarian ideologies among adolescents. Unfortunately, our data did not allow us to capture variations in mothers’ working hours and parents’ division of household work. Classmates’ gender ideologies correlated significantly, yet modestly, with adolescents’ gender ideologies among both native and immigrant female adolescents and among native male adolescents, again with little variation across the four countries. This pattern is in line with a two-way process of acculturation. Classmates are probably less influential than parents because adolescents are not in close contact with all of their classmates, class compositions change every few years, and parents also transmit their beliefs through role modelling, whereas practises in classmates’ homes are usually not observed by other adolescents.

We found only some evidence that countries’ gender and immigration policies and cultures shape gender ideologies of immigrant youth, insofar as Swedish adolescents held the most egalitarian ideologies across the four countries. This finding should be taken with a grain of salt because we cannot exclude that immigrants who endorse more egalitarian values in their origin countries might be more likely to choose Sweden as a destination country. Interestingly, the transmission of gender ideologies from parents, mostly mothers, to adolescents does not vary significantly across the four receiving countries. The similarity in gender ideologies between the parent generation and their adolescent children among both natives and immigrants across all four countries suggests that cohort replacement might not automatically produce further change in the direction of greater egalitarianism in this cohort born in the mid-to-late 1990s. This similarity may be interpreted as evidence of a stalled gender revolution (Goldscheider et al. 2015). However, the results should also be interpreted as reflecting a specific life course phase for adolescents. Additional exploratory analyses for the German subsample of adolescents, for whom we have longitudinal data, point to some degree of subsequent change toward greater egalitarianism between ages 15 and 19. The drivers of such ideological changes during the transition to adulthood will be investigated in future research.

### Limitations and Future Research Directions

Several limitations of our study need to be acknowledged. Recent research on changing gender beliefs in Western post-industrial societies has pointed to the increasing multidimensionality of beliefs (Knight and Brinton 2017) rather than a continuum from traditional to egalitarian ideologies. Due to the limited range of the gender ideology items in the CILS4EU data, we were unable to capture this increasing complexity of beliefs. We recommend that future research consider multiple belief dimensions among native and immigrant groups to provide a more nuanced understanding of the two-way acculturation process. It should also be noted that due to social desirability, parents might tend to provide more egalitarian reports, which might lead us to overestimate intergenerational stability in gender beliefs. Moreover, as a result of measuring gender beliefs only from one parent, mostly mothers, we may underestimate the influence of fathers, especially on sons.

The four receiving countries in our data exhibit some meaningful contextual variations in terms of gender policies and culture, norms of choice, and immigration policies. Yet a sample of just four countries does not allow us to examine the effects of any of these contextual aspects in more detail. For instance, to further explore the recent finding of increasing norms of individual choice concerning gender arrangements in some countries, either a larger country sample or in-depth case studies would make it possible to investigate how immigrant youth navigate differences between such a macro culture that promotes individualism and a micro family culture that may demand more conformity. We would also like to stress that the GEM distance between the origin and receiving countries represents only a proxy measure and cannot fully capture the cultural diversity of various immigrant groups in the four destination countries making up our study.

The cross-sectional nature of the parental data does not allow us to identify causality in the process of belief transmission from parents to children. Furthermore, due to the small share of first-generation immigrants among the adolescents, we were unable to conduct separate analyses for the first and second generation. Unfortunately, the measures of discrimination experiences in the first wave of the CILS4EU data are phrased in a way that does not allow them to be related to any specific aspect of adolescents’ identity (e.g., ethnicity or gender) and therefore does not sufficiently capture variation in discrimination experiences across immigrant groups.

Future research should seek to consider specific discrimination experiences with respect to ethnic or gender identity and resulting internal conflicts as another set of environmental influences that may shape gender ideologies, particularly among immigrant adolescents. Our analysis considered classmates’ average gender ideologies because the allocation of students to classes is usually less selective than adolescents’ choice of their closest friends. However, close friends may have stronger effects on adolescents’ gender beliefs. A promising avenue for future research would be to draw on longitudinal social network data to test two-way models of acculturation by examining the effects of varying friendship networks on changes in natives’ and immigrants’ gender ideologies over time. More generally, further research on acculturation processes among majority groups in Western countries and the effects of immigration policies on natives is needed to critically reflect on the extent to which Western welfare states allow for a multiplicity of values and practices with respect to gender to co-exist.

### Practice Implications

An important finding of the present study from both a policy and practice point of view relates to the great similarity in the intergenerational transmission of gender beliefs across diverse family backgrounds as well as cultural and policy contexts. Parents appear to still be relatively influential in shaping adolescents’ gender ideologies, both through belief transmission as well as through role modelling (e.g., with respect to maternal employment). In several groups, adolescents even report slightly more traditional ideologies than their parents, which may be because paid and unpaid work practices in families are frequently more traditional than reported gender ideologies. Various family policy measures, such as the individual entitlement to well-paid parental leave of moderate length and high-quality, affordable childcare provision (for reviews, see Ferragina 2020; Ray et al. 2010), have been shown to effectively incentivise maternal labour market attachment and fathers’ involvement in childcare. Such measures may therefore indirectly promote more egalitarian ideologies among the next generation.

We also found classmates’ gender ideologies to be predictive of adolescents’ gender beliefs among both immigrants and natives. Because gender ideologies have been found to correlate with competence beliefs and occupational values (van der Vleuten et al. 2016), considering such normative context effects (e.g., in intervention designs) may increase the effectiveness of interventions aimed at strengthening adolescents’ skills and interests in gender-atypical subjects.

### Conclusion

Our study significantly extends existing research by documenting the strong and surprisingly homogenous intergenerational transmission of gender ideologies from parents to adolescents across immigrant and gender groups as well as across four European countries with different normative gender cultures and immigration policy regimes. It also provides novel evidence pointing to significant influences of classmates on adolescents’ gender ideologies, although these relationships appear to be of modest strength compared to those with parents’ beliefs.

## Supplementary Information

ESM 1 (DOCX 53.3 kb)

## References

[CR1] Arends-Tóth J, Van de Vijver FJ (2009). Cultural differences in family, marital, and gender-role values among immigrants and majority members in the Netherlands. International Journal of Psychology.

[CR2] Bolzendahl CI, Myers DJ (2004). Feminist attitudes and support for gender equality: Opinion change in women and men, 1974–1998. Social Forces.

[CR3] Brechwald WA, Prinstein MJ (2011). Beyond homophily: A decade of advances in understanding peer influence processes. Journal of Research on Adolescence.

[CR4] Bussey K, Bandura A (1999). Social cognitive theory of gender development and differentiation. Psychological Review.

[CR5] Carlson DL, Knoester C (2011). Family structure and the intergenerational transmission of gender ideology. Journal of Family Issues.

[CR6] Chang ML (2000). The evolution of sex segregation regimes. American Journal of Sociology.

[CR7] CILS4EU. (2016). *Children of immigrants longitudinal survey in four European. countries. Technical Report. Wave 1–2010/2011, v1.2.0*. Mannheim: Mannheim University. Retrieved from https://www.cils4.eu/images/wave1_material/technical/za5353_technicalreport_wave1.pdf

[CR8] Corrigall EA, Konrad A (2007). Gender role attitudes and careers: A longitudinal study. Sex Roles.

[CR9] Cross WE, Ponterotto JG, Casa JM, Suzuki LA, Alexander CM (1995). The psychology of nigrescence: Revising the cross model. Handbook of multicultural counseling.

[CR10] Crouter AC, Whiteman SD, McHale SM, Osgood DW (2007). Development of gender attitude traditionality across middle childhood and adolescence. Child Development.

[CR11] Cunningham M (2001). The influence of parental attitudes and behaviors on children’s attitudes toward gender and household labor in early adulthood. Journal of Marriage and Family.

[CR12] Cunningham M, Beutel AM, Barber JS, Thornton A (2005). Reciprocal relationships between attitudes about gender and social contexts during young adulthood. Social Science Research.

[CR13] Davis SN, Greenstein TN (2009). Gender ideology: Components, predictors, and consequences. Annual Review of Sociology.

[CR14] Dawson A, Pike A, Bird L (2016). Associations between parental gendered attitudes and behaviours and children’s gender development across middle childhood. European Journal of Developmental Psychology.

[CR15] De Valk HA (2008). Parental influence on work and family plans of adolescents of different ethnic backgrounds in the Netherlands. Sex Roles.

[CR16] De Valk HA, Liefbroer AC (2007). Timing preferences for women’s family-life transitions: Intergenerational transmission among migrants and Dutch. Journal of Marriage and Family.

[CR17] Diehl C, Koenig M, Ruckdeschel K (2009). Religiosity and gender equality: Comparing natives and Muslim migrants in Germany. Ethnic and Racial Studies.

[CR18] Drouhot LG, Nee V (2019). Assimilation and the second generation in Europe and America: Blending and segregating social dynamics between immigrants and natives. Annual Review of Sociology.

[CR19] Dustmann, C., & Frattini, T. (2011). *The socio-economic integration of migrants*. Report by EPolicy Ltd. and Centre for Research and Analysis of Migration (CReAM). Retrieved from https://www.ucl.ac.uk/~uctpb21/reports/Final_report_CLG_06_2011.pdf

[CR20] Farré L, Vella F (2013). The intergenerational transmission of gender role attitudes and its implications for female labour force participation. Economica.

[CR21] Ferragina, E. (2020). Family policy and women’s employment outcomes in 45 high-income countries: A systematic qualitative review of 238 comparative and national studies. *Social Policy & Administration*. Advance online publication. 10.1111/spol.12584

[CR22] Filipp, S.-H., Gerlach, I., Keil, S., Ott, N., & Scheiwe, K. (2016). *Migration und familie: Kindheit mit zuwanderungshintergrund* [*Migration and family: Childhsood with a migration background*]. New York: Springer.

[CR23] Goldscheider F, Goldscheider C, Bernhardt EM (2011). Creating egalitarian families among the adult children of Turkish-and Polish-origin immigrants in Sweden. International Migration Review.

[CR24] Goldscheider F, Bernhardt E, Lappegård T (2015). The gender revolution: A framework for understanding changing family and demographic behavior. Population and Development Review.

[CR25] Grunow D, Veltkamp G, Grunow D, Evertsson M (2016). Institutions as reference points for parents-to-be in European societies: A theoretical and analytical framework. *Couples’ transitions to parenthood*.

[CR26] Heinrich-Böll-Stiftung. (2010)*. Bis in die dritte Generation? Lebensrealitäten junger MigrantInnen. Berlin: Heinrich-Böll-Stiftung [Up to third generation? Life realities of young migrants].* Retrieved from https://heimatkunde.boell.de/sites/default/files/dossier_dritte_generation.pdf

[CR27] Holland JA, de Valk HA (2017). Differences in labour force participation by motherhood status among second-generation Turkish and majority women across Europe. Population Studies.

[CR28] Hurrelmann K, Bauer U (2015). *Einführung in die Sozialisationstheorie [Introduction to socialisation theory]*.

[CR29] Huschek D, Liefbroer AC, de Valk HA (2010). Timing of first union among second-generation Turks in Europe: The role of parents, peers and institutional context. Demographic Research.

[CR30] Idema, H., & Phalet, K. (2007). Transmission of gender-role values in Turkish-German migrant families: The role of gender, intergenerational and intercultural relations. *Zeitschrift für Familienforschung* [*Journal of Family Research*], *19*(1), 71–105. Retrieved from https://nbn-resolving.org/urn:nbn:de:0168-ssoar-58,062

[CR31] Joppke C (2007). Transformation of immigrant integration: Civic integration and antidiscrimination in the Netherlands, France, and Germany. World Politics.

[CR32] Kalter, F., Heath, A. F., Hewstone, M., Jonsson, J. O., Kalmijn, M., Kogan, I., & Van Tubergen, F. (2017a). Children of immigrants longitudinal survey in four European countries (CILS4EU) - Full version. Data file for on-site use. GESIS Data Archive, Cologne. ZA5353 Data file Version 3.3.0. 10.4232/cils4eu.5353.3.3.0

[CR33] Kalter, F., Heath, A. F., Hewstone, M., Jonsson, J. O., Kalmijn, M., Kogan, I., & Van Tubergen, F. (2017b). Children of immigrants longitudinal survey in four European countries (CILS4EU) - Reduced version. Reduced data file for download and off-site use. GESIS Data Archive,Cologne, ZA5656 Data file Version 3.3.0. 10.4232/cils4eu.5656.3.3.0.

[CR34] Katz PA, Ksansnak KR (1994). Developmental aspects of gender role flexibility and traditionality in middle childhood and adolescence. Developmental Psychology.

[CR35] Knight CR, Brinton MC (2017). One egalitarianism or several? Two decades of gender-role attitude change in Europe. American Journal of Sociology.

[CR36] Koopmans R (2013). Multiculturalism and immigration: A contested field in cross-national comparison. Annual Review of Sociology.

[CR37] Kremer M (2007). *How welfare states care: Culture, gender and parenting in Europe*.

[CR38] Kretschmer D (2018). Explaining differences in gender role attitudes among migrant and native adolescents in Germany: Intergenerational transmission, religiosity, and integration. Journal of Ethnic and Migration Studies.

[CR39] Laftman SB (2008). Parent presence and gender-typicalness of educational choice. The British Journal of Sociology.

[CR40] Lee EAE, Troop-Gordon W (2011). Peer processes and gender role development: Changes in gender atypicality related to negative peer treatment and children’s friendships. Sex Roles.

[CR41] Logan JR, Shin HJ (2012). Assimilation by the third generation? Marital choices of White ethnics at the dawn of the twentieth century. Social Science Research.

[CR42] Norris P, Inglehart RF (2012). Muslim integration into Western cultures: Between origins and destinations. Political Studies.

[CR43] OECD. (2014.) *Finding the way: A discussion of the Swedish migration integration system. Paris.* Retrieved from https://www.oecd.org/migration/swedish-migrant-intergation-system.pdf

[CR44] OECD. (2015). *The ABC of gender equality in education: Aptitude, behaviour, confidence.* Retrieved from https://www.oecd.org/pisa/keyfindings/pisa-2012-results-gender-eng.pdf

[CR45] Pels T, Nijsten C, Hagendoorn L, Veenman J, Vollebergh W (2003). Myths and realities of diversity in parenting and parent-child relations: A comparison of indigenous and non-indigenous families in the Netherlands. *Integrating immigrants in the Netherlands: Cultural versus socio-economic integration*.

[CR46] Phalet K, Schönpflug U (2001). Intergenerational transmission of collectivism and achievement values in two acculturation contexts: The case of Turkish families in Germany and Turkish and Moroccan families in the Netherlands. Journal of Cross-cultural Psychology.

[CR47] Phinney JS, Ong AD (2007). Conceptualization and measurement of ethnic identity: Current status and future directions. Journal of Counseling Psychology.

[CR48] Platt L, Polavieja J (2016). Saying and doing gender: Intergenerational transmission of attitudes towards the sexual division of labour. European Sociological Review.

[CR49] Prinstein MJ, Dodge KA (2008). *Understanding peer influence in children and adolescents*.

[CR50] Ray R, Gornick JC, Schmitt J (2010). Who cares? Assessing generosity and gender equality in parental leave policy designs in 21 countries. Journal of European Social Policy.

[CR51] Risman BJ (2004). Gender as a social structure: Theory wrestling with activism. Gender & Society.

[CR52] Röder A (2014). Explaining religious differences in immigrants’ gender role attitudes: The changing impact of origin country and individual religiosity. Ethnic and Racial Studies.

[CR53] Röder A, Mühlau P (2014). Are they acculturating? Europe’s immigrants and gender egalitarianism. Social Forces.

[CR54] Stahl JF, Schober PS (2018). Convergence or divergence? Educational discrepancies in work-care arrangements of mothers with young children in Germany. Work, Employment and Society.

[CR55] Stevenson MR, Black KN (1988). Paternal absence and sex-role development: A meta-analysis. Child Development.

[CR56] Supple AJ, Ghazarian SR, Frabutt JM, Plunkett SW, Sands T (2006). Contextual influences on Latino adolescent ethnic identity and academic outcomes. Child Development.

[CR57] Tenenbaum HR, Leaper C (2002). Are parents’ gender schemas related to their children’s gender-related cognitions? A meta-analysis. Developmental Psychology.

[CR58] Umana-Taylor AJ, Guimond AB (2010). A longitudinal examination of parenting behaviors and perceived discrimination predicting Latino adolescents’ ethnic identity. Developmental Psychology.

[CR59] Umaña-Taylor AJ, Yazedjian A, Bámaca-Gómez M (2004). Developing the ethnic identity scale using Eriksonian and social identity perspectives. Identity: An International Journal of Theory and Research.

[CR60] van der Vleuten M, Jaspers E, Maas I, van der Lippe T (2016). Boys’ and girls’ educational choices in secondary education. The role of gender ideology. Educational Studies.

[CR61] Wang S (2019). The role of gender role attitudes and immigrant generation in ethnic minority women’s labor force participation in Britain. Sex Roles.

[CR62] Ward C, Geeraert N (2016). Advancing acculturation theory and research: The acculturation process in its ecological context. Current Opinion in Psychology.

[CR63] West C, Zimmerman DH (1987). Doing gender. Gender & Society.

[CR64] Wimmer A, Soehl T (2014). Blocked acculturation: Cultural heterodoxy among Europe’s immigrants. American Journal of Sociology.

