# Supplementary material for: Socialisation Influences on Gender Ideologies of Immigrant and Native Youth in Germany, England, Sweden and the Netherlands
Source: Sex Roles. 2020 Dec 6;85(3-4):113–27. doi: 10.1007/s11199-020-01208-z (PMC7719056; doi:10.1007/s11199-020-01208-z)
Supplement: Supplementary file 1 — (DOCX 53.3 kb) [file 11199_2020_1208_MOESM1_ESM.docx]

Online supplement Guerrero, L. S., and Schober, P. S. (2020). Socialisation influences on gender ideologies of immigrant and native youth in Germany, England, Sweden, and the Netherlands. *Sex Roles*. Laia Sánchez Guerrero, University of Tübingen. Email: laia.sanchez-guerrero@uni-tuebingen.de

| Table 1s | | | | | | | | |  |
| --- | --- | --- | --- | --- | --- | --- | --- | --- | --- |
| *Distribution of answers. Items for gender ideology factor* | | | | | | | | |  |
|  | Immigrants | | |  | Natives | | | | |
| *In a family, who should do the following:* | Adolescents |  | Parents |  | | Adolescents |  | Parents | |
|  | *n* (%) |  | *n* (%) |  | | *n* (%) |  | *n* (%) | |
| *Take care of children* |  |  |  |  | |  |  |  | |
| Mostly the woman | 359 (22) |  | 262 (16) |  | | 685 (16) |  | 557 (13) | |
| Mostly the man or both the same | 1276 (78) |  | 1374 (84) |  | | 3596 (84) |  | 3724 (87) | |
| *Cook* |  |  |  |  | |  |  |  | |
| Mostly the woman | 802 (49) |  | 775 (45) |  | | 1413 (33) |  | 1070 (25) | |
| Mostly the man or both the same | 834 (51) |  | 900 (55) |  | | 2868 (67) |  | 3122 (75) | |
| *Earn money* |  |  |  |  | |  |  |  | |
| Mostly the man | 654 (40) |  | 507 (31) |  | | 1284 (30) |  | 942 (22) | |
| Mostly the woman or both the same | 982 (60) |  | 1129 (69) |  | | 2997 (70) |  | 3339 (78) | |
| *Clean the house* |  |  |  |  | |  |  |  | |
| Mostly the woman | 638 (39) |  | 556 (34) |  | | 1413 (33) |  | 899 (21) | |
| Mostly the man or both the same | 998 (61) |  | 1080 (66) |  | | 2868 (67) |  | 3382 (79) | |

*Note*. Source: CILS4EU – Full version (ZA5353 data file version 3.3.0).

| Table 2s |  | |  | | |  |  | | |  |
| --- | --- | --- | --- | --- | --- | --- | --- | --- | --- | --- |
| *Descriptive statistics of control variables (weighted)* | | | | | | | | |  |  |
|  |  | Immigrants | |  | Natives | | |  |  |  |
| Variables |  | Mean (SD) or *n* (%) | |  | Mean (SD) or *n* (%) | | |  |  |  |
| Adolescent’s contact with natives |  | 3.12 (.97) | |  | - | | |  |  |  |
| Adolescent’s proportion of native friends^a^ |  |  | |  | - | | |  |  |  |
| None or a few |  | 524 (32) | |  | - | | |  |  |  |
| Half or a lot |  | 785 (48) | |  | - | | |  |  |  |
| All or almost all |  | 327 (20) | |  | - | | |  |  |  |
| Parent’s contact with natives |  | 2.85 (.91) | |  |  | | |  |  |  |
| Parent’s proportion of native friends^a^ |  |  | |  |  | | |  |  |  |
| None or a few |  | 605 (37) | |  | - | | |  |  |  |
| Half or a lot |  | 785 (48) | |  | - | | |  |  |  |
| All or almost all |  | 246 (15) | |  | - | | |  |  |  |
| Immigrant background |  |  | |  |  | | |  |  |  |
| First generation |  | 327 (20) | |  | - | | |  |  |  |
| Second generation |  | 1309 (80) | |  | - | | |  |  |  |
| Religiosity |  | 2.74 (1.23) | |  | 1.85 (.92) | | |  |  |  |
| Religious denomination |  |  | |  |  | | |  |  |  |
| No religion |  | 245 (15) | |  | 1755 (41) | | |  |  |  |
| Christianity |  | 605 (37) | |  | 2397 (56) | | |  |  |  |
| Islam |  | 622 (38) | |  | - | | |  |  |  |
| Other |  | 164 (10) | |  | 129 (3) | | |  |  |  |
| Child's gender |  |  | |  |  | | |  |  |  |
| Female |  | 785 (48) | |  | 2098 (49) | | |  |  |  |
| Male |  | 851 (52) | |  | 2183 (51) | | |  |  |  |
| Parent's gender |  |  | |  |  | | |  |  |  |
| Female |  | 1145 (70) | |  | 3510 (82) | | |  |  |  |
| Male |  | 491 (30) | |  | 751 (18) | | |  |  |  |
| Father employed |  |  | |  |  | | |  |  |  |
| No |  | 180 (11) | |  | 171 (4) | | |  |  |  |
| Yes |  | 1456 (89) | |  | 4110 (96) | | |  |  |  |
| Mother’s education |  |  | |  |  | | |  |  |  |
| Less than university |  | 1325 (81) | |  | 3382 (79) | | |  |  |  |
| University degree |  | 311 (19) | |  | 899 (21) | | |  |  |  |
| Father's education |  |  | |  |  | | |  |  |  |
| Less than university |  | 1292 (79) | |  | 3339 (78) | | |  |  |  |
| University degree |  | 344 (21) | |  | 942 (22) | | |  |  |  |
| Household size |  | 4.73 (1.26) | |  | 4.36 (1.04) | | |  |  |  |

*Note*. Source: CILS4EU – Full version (ZA5353 data file version 3.3.0).

^a^Source: CILS4EU- Reduced version (ZA5656 data file Version 3.3.0).

Table 2s continued

| *Descriptive statistics of control variables (weighted)* | | | | | |
| --- | --- | --- | --- | --- | --- |
|  |  | Immigrant |  | Native |  |
| Variables |  | Mean (SD) or *n* (%) |  | Mean (SD) or *n* (%) |  |
| Family structure |  |  |  |  |  |
| Two parents |  | 1571 (96) |  | 4024 (94) |  |
| Step-family |  | 59 (3) |  | 214 (5) |  |
| Single-parent |  | 15 (1) |  | 43 (1) |  |
| Has at least a sister |  |  |  |  |  |
| Yes |  | 916 (56) |  | 2226 (52) |  |
| No |  | 720 (44) |  | 2055 (42) |  |
| Has at least a brother |  |  |  |  |  |
| Yes |  | 982 (60) |  | 2355 (55) |  |
| No |  | 654 (40) |  | 1926 (45) |  |
| Share of females in class |  | 48 (18) |  | 50 (19) |  |
| Share of immigrants in school |  |  |  |  |  |
| 0-10% |  | 144 (9) |  | 1062 (25) |  |
| 10-30% |  | 471 (29) |  | 1875 (44) |  |
| 30-60% |  | 471 (29) |  | 976 (23) |  |
| 60-100% |  | 520 (32) |  | 291 (7) |  |
| Independent schools |  | 30 (2) |  | 77 (2) |  |

*Note*. Source: CILS4EU – Full version (ZA5353 data file version 3.3.0)

| Table 3s  *Control variables for OLS Regression for Adolescents’ Gender Ideologies by Gender and Immigrant Background* | | | | | | | | | | | | | | | | | | | | | | | | |  |
| --- | --- | --- | --- | --- | --- | --- | --- | --- | --- | --- | --- | --- | --- | --- | --- | --- | --- | --- | --- | --- | --- | --- | --- | --- | --- |
|  |  | | Immigrant | | | | | | | | | |  | | Native | | | | | | | | | |  |
|  |  | | Female (n=842) | | | |  | | Male (n= 794) | | | |  | | Female (n=2,169) | | | |  | | Male (n=2,112) | | | |  |
| Control variables | | | *b* | | 95% CI | |  | | *b* | | 95% CI | |  | | *b* | | 95% CI | |  | | *b* | | 95% CI | |  |
| Father employed | | | -.24* | | [-.44, .04] | |  | | -.06 | | [-.28, .16] | |  | | -.32*** | | [-.47, -.18] | |  | | -.20 | | [-.41, .00] | |  |
| University-educated mother | | | .15 | | [-.02, .32] | |  | | .11 | | [-.08, .31] | |  | | .15** | | [.06, .25] | |  | | .16** | | [.03, .29] | |  |
| University-educated father | | | .03 | | [-.15, .21] | |  | | -.01 | | [-.20, .17] | |  | | .04 | | [-.05, .14] | |  | | .09 | | [-.02, .21] | |  |
| Adolescent’s contact with natives^a^ | | | .03 | | [-.03, .10] | |  | | .09** | | [0.26, .16] | |  | | - | | - | |  | | - | | - | |  |
| \| Parent’s contact with natives^a^ \|  \| -0.00 \| [-.06,.05] \|  \| 0.05 \| [-.02, .12] \|  \| - \| - \|  \| - \| - \| \| --- \| --- \| --- \| --- \| --- \| --- \| --- \| --- \| --- \| --- \| --- \| --- \| --- \| | |  | | -.00 | | [-.06,.05] | |  | | .05 | | [-.02, .12] | |  | | - | | - | |  | | - | | - | |
| Second-generation immigrant | |  | | .07 | | [-.07, .21] | |  | | -.09 | | [-.25, .06] | |  | | - | | - | |  | | - | | - | |
| Religion  (Ref. No religion) | |  | |  | |  | |  | |  | |  | |  | | - | | - | |  | | - | | - | |
| Christianity | | | -.05 | | [-.25, .14] | |  | | -.07 | | [-.32, 17] | |  | | -.01 | | [-.10, .08] | |  | | -.06 | | [-.17, .03] | |  |
| Islam |  | | .02 | | [-.21, .25] | |  | | -.07 | | [-.35, .20] | |  | | - | | - | |  | | - | |  | |  |
| Other |  | | .09 | | [-.15, .35] | |  | | -.11 | | [-.42, .19] | |  | | .02 | | [-.31, .27] | |  | | .12 | | [-.13, .38] | |  |
| Religiosity^a^ | | | -.08* | | [-.15, -.01] | |  | | .01 | | [-.07, .10] | |  | | -.06** | | [-.12, -.01] | |  | | -.01 | | [-.07, .05] | |  |
| Family structure  (Ref. Two parents) | | |  | |  | |  | |  | |  | |  | |  | |  | |  | |  | |  | |  |
| Step-family | | | .09 | | [-.33, -.14] | |  | | .02 | | [-.22, .26] | |  | | .09 | | [-.09, .27] | |  | | .06 | | [-12, .26] | |  |
| Single parent | | | .06 | | [-.38, .51] | |  | | .20 | | [-.21, .62] | |  | | .02 | | [-.29, .33] | |  | | .33* | | [.03, .64] | |  |
| Sister dummy | | | .05 | | [-.08, .18] | |  | | .05 | | [-.10, .21] | |  | | -.07 | | [-.17, .02] | |  | | .03 | | [-.06, .14] | |  |
| Brother dummy | | | .09 | | [-.05, .25] | |  | | .13 | | [-.01, .28] | |  | | -.07* | | [-.16, .01] | |  | | -.05 | | [-.16, .05] | |  |
| Household size | | | -.03 | | [-.10, .02] | |  | | -.06* | | [-.13, -.04] | |  | | .02 | | [-.01, .07] | |  | | -.01 | | [-.07, .04] | |  |
| Respondent mother (Ref. Father) | | | .01 | | [-.11, .14] | |  | | .04 | | [-.10, .19] | |  | | -.06 | | [-.16, .03] | |  | | -.08 | | [-.18, .02] | |  |

*Note.* Z-transformations of factor variables; Ref.= Reference category; 95% CI= 95% Confidence Intervals. Source: CILS4EU – Full version (ZA5353 data file version 3.3.0).

^a^Higher values indicate more egalitarian ideologies.

**p* < .05. ** *p* < .01. *** *p* < .001.

(Table 3s continues on the next page)

Table 3s continued

| *Control variables for OLS Regression for Adolescents’ Gender Ideologies by Gender and Immigrant Background* | | | | | | | | | | | | |
| --- | --- | --- | --- | --- | --- | --- | --- | --- | --- | --- | --- | --- |
|  |  | Immigrant | | | | |  | Native | | | | |
|  |  | Female (*n* = 842) | |  | Male (*n* = 794) | |  | Female (*n* = 2169) | |  | Male (*n* = 2112) | |
| Variables | | *b* | 95% CI |  | *b* | 95% CI |  | *b* | 95% CI |  | *b* | 95% CI |
| Share of immigrants in school | |  |  |  |  |  |  |  |  |  |  |  |
| (Ref. less than 10%) | |  |  |  |  |  |  |  |  |  |  |  |
| 10-30% |  | -.05 | [-.23,.11] |  | .05 | [-.20, .31] |  | -.01 | [.10, .07] |  | .01 | [-.09, -12] |
| 30-60% |  | -.15 | [-.34, .24] |  | .15 | [-.10, .42] |  | -.01 | [-.11, .08] |  | .07 | [-.04, .18] |
| 60-100% | | -.13 | [-.33, .06] |  | .19 | [-.53, .78] |  | -.00 | [-.15, .17] |  | .25** | [.07, .44] |
| Independent schools | | -.10 | [-.61, .40] |  | .12 | [-.53, .78] |  | .13 | [-15, .12] |  | .30 | [-.32, .92] |
| Share of females in class | | -.01 | [-.42, .49] |  | .12 | [-.32, .57] |  | -.08 | [-.29, .12] |  | -.11 | [-.38, .15] |
| Constant |  | .57* | [.07, 1.08] |  | .15 | [-.36, .67] |  | .44*** | [.15, .74] |  | .03 | [-.36, .42] |
| R-Squared | | .27 |  |  | .24 |  |  | .26 |  |  | .20 |  |

*Note*. Z-transformations of factor variables; Ref.= Reference category; 95% CI= 95% Confidence Intervals. Source: CILS4EU – Full version (ZA5353 data file version 3.3.0)

**p* < .05. ** *p* < .01. *** *p* < .001.

| Table 4s | | | | | | |  |
| --- | --- | --- | --- | --- | --- | --- | --- |
| *Robustness checks: OLS Regression for Adolescents’ Gender Ideologies by Immigrant Background with Bonferroni corrections* | | | | | | |  |
|  |  | Immigrant (n=1,555) | |  | Native (n=4,281) | | |
| Variables | | *b* | 95% CI |  | *b* | 95% CI | |
| Parental gender ideology^a^ | | .24*** | [.19, .30] |  | .25*** | [.21, .28] | |
| Class gender ideology^a^ | | .07* | [.02, .12] |  | .09*** | [.06, .12] | |
| Female |  | .31*** | [.22, .41] |  | .41*** | [.35, .47] | |
| Mother Employment | | .25*** |  |  | .32*** | [.23, .41] | |
| Gender empowerment distance | |  |  |  |  |  | |
| (Ref. Small) | |  |  |  |  |  | |
| Medium |  | -.10 | [-.22, .02] |  | - | - | |
| Large |  | -.13 | [-.28, 0.2] |  | - | - | |
| Destination country | |  |  |  |  |  | |
| (Ref. Sweden) | |  |  |  |  |  | |
| England |  | -.12 | [-.32, .09] |  | -.18** | [-.28, .08] | |
| Germany | | -.31*** | [-.46, -.16] |  | -.29*** | [-.38, -.20] | |
| Netherlands | | -.33** | [.52, .15] |  | -.34*** | [-.45, .24] | |
| **Control variables** | |  |  |  |  |  | |
| Father employed | | -.15 | [-.31, .01] |  | -.27*** | [-.39, -.14] | |
| University-educated mother | | .16* | [.02, .29] |  | .16*** | [.08, .24] | |
| University-educated father | | -.00 | [-.14, .14] |  | .06 | [-.02, .14] | |
| Adolescent: proportion of native friends  (Ref. None or a few) |  |  |  |  |  |  | |
| Half or a lot |  | .18** | [.06, .30] |  | - | - | |
| All or almost all |  | .14 | [-.02, .31] |  | - | - | |
| Parent: proportion of native friends  (Ref. None or a few) |  |  |  |  |  |  | |
| Half or a lot |  | .05 | [-.06, .15] |  | - | - | |
| All or almost all |  | .02 | [-.12, .16] |  | - | - | |
| Second-generation Immigrant |  | .01 | [-.11, .12] |  |  |  | |
| Religion  (Ref. No religion) |  |  |  |  |  |  | |
| Christianity | | -.13 | [-.29, .02] |  | -.04 | [-.11, .03] | |
| Islam |  | -.06 | [-.24, .12] |  | - | - | |
| Other |  | -.09 | [-.29, .12] |  | .08 | [-.09, .26] | |
| Religiosity^a^ | | -.02 | [-.08, .04] |  | .04 | [-.08, .00] | |
| Family structure  (Ref. Two parents) | |  |  |  |  |  | |
| Step-family | | .13 | [-.11, .38] |  | .08 | [-.05, .21] | |
| Single Parent | | -.06 | [-.56, .43] |  | .15 | [-.06, .27] | |

*Note*. Z-transformations of factor variables; Ref.= Reference category; 95% CI= 95% Confidence Intervals. Source: CILS4EU- Reduced version (ZA5656 data file Version 3.3.0).

^a^Higher values indicate more egalitarian ideologies.

**p* < .05. ** *p* < .01. *** *p* < .001.

Table 4s continued

| *Robustness checks: OLS Regression for Adolescents’ Gender Ideologies by Immigrant Background with Bonferroni corrections* | | | | | | |
| --- | --- | --- | --- | --- | --- | --- |
|  |  | Immigrants (n=1555) | |  | Natives (n=4281) | |
| Control variables | | *b* | 95% CI |  | *b* | 95% CI |
| Sister dummy | | .10 | [-.01, .20] |  | -.02 | [-.09, .05] |
| Brother dummy | | .15** | [.05, .26] |  | -.06 | [-.13, .00] |
| Household size | | -.06** | [-.10, -.02] |  | .00 | [-.03, .04] |
| Respondent mother | | .016 | [-.09, .12] |  | -.11* | [-.27, -.05] |
| Share of immigrants in school | |  |  |  |  |  |
| (Ref. less than 10%) | |  |  |  |  |  |
| 10-30% |  | -.07 | [-.23, .08] |  | .00 | [-.06, .07] |
| 30-60% |  | -.09 | [-.24, .08] |  | .03 | [-.04, .11] |
| 60-100% | | -.16 | [-.18, .15] |  | .13* | [.00, .27] |
| Independent schools | | -.04 | [-.40, .32] |  | .19 | [-.03, .41] |
| Share of females in class | | -.06 | [-.36, .23] |  | -.11 | [-.27, .06] |
| Constant |  | .06 | [-.33, .44] |  | .05 | [-.18, .27] |
| R-Squared | | .26 | |  | .26 | |

*Note*. Z-transformations of factor variables; Ref.= Reference category; 95% CI= 95% Confidence Intervals. CILS4EU- Reduced version (ZA5656 data file Version 3.3.0).

**p* < .05. ** *p* < .01. *** *p* < .001.

| Table 5s | | |  | |  | |  | |  | | |
| --- | --- | --- | --- | --- | --- | --- | --- | --- | --- | --- | --- |
| *Robustness check: Main effects of key variables and interactions with gender, by immigrant background* | | | | | | | | | | |  |
|  | Immigrants (n=1,555) | | |  | | Natives  (n=4,281) | | | |  |  |
| Variables and interactions | *b* | 95% CI | |  | | *b* | | 95% CI | |  |  |
| Parental gender ideology^a^ | .23*** | [.15, .31] | |  | | .24*** | | [.19, .28] | |  |  |
| Female (Ref. Male) | .32*** | [.23, .42] | |  | | .41*** | | [.35, .46] | |  |  |
| **Interaction** |  |  | |  | |  | |  | |  |  |
| Parental gender ideology^a^ x female (Ref. Male) | .03 | [-.06, .12] | |  | | .02 | | [-.03, .08] | |  |  |
| Constant | .05 | [-.32, .43] | |  | | .05 | | [-.18, .27] | |  |  |
| R-Square | .26 | | |  | | .26 | | | |  |  |
| Class gender ideology^a^ | .05 | [-.01, .11] | |  | | .09*** | | [.05, .13] | |  |  |
| Female (Ref. Male) | .34** | [.24, .44] | |  | | .41*** | | [.35, .47] | |  |  |
| **Interaction** |  |  | |  | |  | |  | |  |  |
| Class gender ideology x female (Ref. Male) | .04 | [-.03, .10] | |  | | .00 | | [-.04, .04] | |  |  |
| Constant | .05 | [-.33, .43] | |  | | .05 | | [-.18, .27] | |  |  |
| R-Square | .26 | | |  | | .26 | | | |  |  |
| Mother is employed (ref. no) | .24*** | [.08, .40] | |  | | .29*** | | [.18, .40] | |  |  |
| Female (Ref. Male) | .34*** | [.18, .50] | |  | | .47*** | | [.30, .64] | |  |  |
| **Interaction** |  |  | |  | |  | |  | |  |  |
| Mother is employed (Ref. not employed) x female (Ref. Male) | -.04 | [-.24, .17] | |  | | .07 | | [-.24, .11] | |  |  |
| Constant | .05 | [-.33, .44] | |  | | .02 | | [-.23, .26] | |  |  |
| R-Square | 0.26 | | |  | | .26 | | | |  |  |
| Parental gender ideology^a^ | .23*** | [.14, .33] | |  | | .30*** | | [.24, .37] | |  |  |
| Respondent mother (Ref. Father) | .02 | [-.08, .12] | |  | | -.07* | | [-.14, -.00] | |  |  |
| **Interaction** |  |  | |  | |  | |  | |  |  |
| Parental gender ideology^a^ x parent gender (Ref. Father) | .01 | [-.09, -12] | |  | | -.07 | | [-.14, .00] | |  |  |
| Constant | .06 | [-.32, .43] | |  | | .04 | | [-.18, .27] | |  |  |
| R-Square | .26 | | |  | | .26 | | | |  |  |

*Note*. Z-transformations of factor variables; Ref.= Reference category; 95% CI= 95% Confidence Intervals. Source: CILS4EU- Reduced version (ZA5656 data file Version 3.3.0).

^a^Higher values indicate more egalitarian ideologies.

**p* < .05. ** *p* < .01. *** *p* < .001.

| Table 6s | | | | | | | |
| --- | --- | --- | --- | --- | --- | --- | --- |
| *Robustness checks: Main Effects of Parental Gender Ideology and Interactions for Immigrant Adolescents with Bonferroni corrections* | | | | | | | |
|  | |  | |  | | Immigrants (n=1,555) | |
| Variables | | | |  | | *b* | 95% CI |
| ***Hypothesis 3a/b.*** *Interaction of parental gender ideology and similarity of gender culture* | | | | | | | |
| Parental gender ideology^a^ | | | |  | | .30*** | [.21, .39] |
| Female (Ref. Male) |  |  |  |  |  | .31*** | [.22, .41] |
| Distance in gender empowerment  (Ref. Small) | | | |  | |  | |
| Medium | |  | |  | | -.10 | [-.21, .02] |
| Large | |  | |  | | -.14 | [-.30, .01] |
| **Interaction** | | | |  | |  | |
| Parental gender ideology^a^ x distance in gender empowerment  (Ref. Small) | | | | | | | |
| PGI x Medium | |  | |  | | -.06 | [-.19, -.07] |
| PGI x Large | |  | |  | | -.09 | [-.20, .02] |
| Constant | |  | |  | | .06 | [-.32, .43] |
| R-Square | |  | |  | | .26 | |
| ***Hypothesis 5.*** *Among immigrants, parents’ gender ideologies are less strongly associated with adolescents’ ideologies in Sweden than in Germany, England and the Netherlands.* | | | | | | | |
| Parental gender ideology^a^ | | |  | | .17* | | [.03, .30] |
| Female (Ref. Male) |  |  |  |  |  | .31*** | [.22, .40] |
| Destination country | | |  | |  | |  |
| (Ref. Sweden) | | |  |  |  | |  |
| England |  | |  | | -.10 | | [-.30, .10] |
| Germany | | |  | | -.33*** | | [-.49, - .18] |
| Netherlands | | |  | | -.34*** | | [-.53, .16] |
| **Interaction** | | |  | |  | |  |
| Parental gender ideology^a^ x destination country  (Ref: Sweden) | | | | | | | |
| PGI x England | | |  | | .17 | | [-.03, .37] |
| PGI x Germany | | |  | | .07 | | [-.08, .22] |
| PGI x Netherlands | | |  | | .05 | | [-.13, .23] |
| Constant |  | |  | | .04 | | [-.32, .41] |
| R-Squared | | |  | | .26 | | |

*Note*. Z-transformations of factor variables; Ref.= Reference category; 95% CI= 95% Confidence Intervals. Source: CILS4EU- Reduced version (ZA5656 data file Version 3.3.0).

^a^Higher values indicate more egalitarian ideologies.

**p* < .05. ** *p* < .01. *** *p* < .00
